# Aerobic exercise-based cardiac rehabilitation in Chinese patients with coronary heart disease: study protocol for a pilot randomized controlled trial

**DOI:** 10.1186/s13063-018-2771-8

**Published:** 2018-07-09

**Authors:** Richard Y. Cao, Hongchao Zheng, Qiongyao Mi, Qing Li, Wenchao Yuan, Yueyou Ding, Jian Yang

**Affiliations:** The Joint Laboratory of Cardiac Rehabilitation, Shanghai Xuhui Central Hospital & Shanghai University, 966 Middle Huaihai Road, 200031 & 99 Shangda Road, Shanghai, 200444 China

**Keywords:** Coronary heart disease, Cardiac rehabilitation, Aerobic metabolism, Biomarker, Protocol, Randomization

## Abstract

**Background:**

Cardiovascular disease is the leading cause of morbidity and mortality in the world, including China. Cardiac rehabilitation (CR) has been demonstrated to be beneficial in reducing cardiovascular mortality, myocardial infarction, and cerebrovascular events. This pilot study seeks to assess the feasibility of aerobic-exercise-based CR in Chinese patients with coronary heart disease (CHD) and outcomes of aerobic metabolism capacity and molecular biomarkers.

**Methods/design:**

This study is a single-center, pilot, randomized, controlled study that is currently being carried out at a regional hospital in Shanghai. Forty patients with CHD who underwent percutaneous coronary intervention will be randomly allocated into either the intervention group or control group. Participants in the intervention group will undergo 8 weeks of aerobic exercise with targeted intensity and participants in the control group will undergo 8 weeks of leisure exercise. The primary measurement is the feasibility of the trial; the secondary measurement is the capacity of aerobic metabolism and the exploratory measurement includes additional molecular biomarkers underlying cardiovascular function.

**Discussion:**

This is the first prospective randomized and controlled clinical study in China that assesses the parameters of aerobic metabolism and comprehensively screens for substantial blood biomarkers to reveal the molecular mechanisms underlying changes in cardiovascular function after aerobic exercise with targeted intensity in participants with CHD. The success of this study will contribute to guide the design of future CR studies in patients with CHD in China.

**Trial registration:**

Chinese Clinical Trial Registry, ChiCTR-IPR-17010556. Registered on 1 June 2016.

**Electronic supplementary material:**

The online version of this article (10.1186/s13063-018-2771-8) contains supplementary material, which is available to authorized users.

## Background

Cardiovascular disease (CVD) is the leading cause of morbidity and mortality in the world [1]. It is not only the top cause of morbidity but also accounts for more than 40% of all deaths because of the continuous increase in the incidence of CVD due to lifestyle changes, urbanization, and an accelerated rate of aging after rapid economic growth over the past 30 years in China [2, 3]. The rising number of CVD deaths in China is largely due to the significant increase in ischemic heart disease, also known as coronary heart disease (CHD), which has been shown to be preventable [2, 4]. Therefore, prevention and control of CHD in China, a country with almost one fifth of the world’s population, may have a significant impact on efforts to achieve sustainable global reductions in CVD rates.

Cardiac rehabilitation (CR), a multifaceted intervention program, has been shown to reduce cardiovascular mortality, myocardial infarction, and cerebrovascular events [5]. It is designed to help patients with CVD achieve optimal physical, psychological, and social status. CR helps to stabilize, slow, or even reverse the progression of the underlying atherosclerotic processes, thereby reducing morbidity and mortality [6, 7]. A large study involving over 600,000 American patients with coronary disease showed that participants undergoing high-dose CR (25 or more sessions) had a lower mortality rate than those undergoing low-dose CR (1–24 sessions) within 5 years of discharge from hospital [8]. CR has been shown to be the third most cost-effective intervention after aspirin and beta blockers to reduce cardiovascular mortality [9]. Moreover, a recent study showed that 3 months of CR, which served as a non-pharmacological way to inhibit platelets, significantly decreased platelet hyperactivity [10].

Although the optimal exercise characteristics (type, dosage, and intensity) that yield the most beneficial effects in patients with CHD are still controversial [11], growing evidence suggests that higher intensity interval training is more effective than lower intensity continuous training for improving aerobic capacity in patients with CHD [12]. This finding is consistent with our previous findings that aerobic exercise with targeted intensity can improve aerobic metabolic capacity in patients who have had a stroke [13]. Markers of aerobic metabolism along with other cardiovascular biomarkers such as C-reactive protein, brain natriuretic peptide, and vascular endothelial growth factor B are important predictors of cardiovascular health or risk [14–16]. Nevertheless, changes in cardiovascular biomarkers after CR have never been investigated comprehensively.

Here, we designed a pilot, randomized, controlled trial to assess the feasibility of aerobic-exercise-based CR on aerobic metabolic capacity and substantial cardiovascular biomarkers in patients with CHD after percutaneous coronary intervention (PCI). The completion of this pilot study will address aspects of feasibility and key issues of uncertainty to promote future pivotal research in generalizing the CR program in China.

## Methods/design

### Aim

The purpose of this pilot study is to test the feasibility of testing the effect of aerobic-exercise-based CR on aerobic capacity and underlying cardiovascular function expressed by molecular biomarkers in patients with CHD after PCI.

### Design

This pilot study is a single-center, prospective, randomized, and controlled clinical trial that will be carried out in the Department of Rehabilitation, Shanghai Xuhui Central Hospital, between 1 June 2016 and 31 June 2018.

All aspects of the study design and protocol adhere to Standard Protocol Items: Recommendations for Interventional Trials (SPIRIT) guidelines (Additional file 1) [17] and will be reported according to the Consolidated Standards of Reporting Trials (CONSORT) statement (Additional file 2) [18]. The protocol has been reviewed and approved by the Ethics Committee of Shanghai Xuhui Central Hospital (approval number 2016–10). The research team of this study will ensure that the ethics standards are adhered to during the study procedure and that they are in accordance with The Code of Ethics of the World Medical Association (Declaration of Helsinki). Participation will be voluntary, and participants can withdraw from the study at any time without any negative consequences. Written consent forms (Additional file 3) and data collected from participants will be kept anonymous and confidential.

### Participant recruitment

A booklet containing basic information about aerobic-exercise-based CR (Table 1) will be distributed to patients registered for PCI in Shanghai Xuhui Central Hospital. Eligible patients who meet the inclusion criteria will be recruited from the Department of Cardiology at the time of discharge after PCI to participate in the CR program conducted in the Department of Rehabilitation.

**Table 1:** A booklet containing information on cardiac rehabilitation

List of information related to cardiac rehabilitation	
∙ What cardiac rehabilitation is	
∙ Why patients with cardiovascular diseases need cardiac rehabilitation	
∙ What cardiopulmonary exercise testing is	
∙ Who can take part in the aerobic exercise-based cardiac rehabilitation	
∙ What individualized exercise prescription is	
∙ A brief introduction to the Department of Rehabilitation in Shanghai Xuhui Central Hospital	

The inclusion criteria are as follows: (1) diagnosis of CHD by a cardiologist based on the combination of clinical symptoms and findings on electrocardiogram and/or echocardiogram and/or coronary angiogram; (2) age between 45 and 80 years; (3) low to moderate risk of cardiovascular event posed by participating in CR based on the Chinese cardiac risk stratification as determined by the experts [19]; and (4) provision of a signed consent form. Strict exclusion criteria are used to avoid unexpected adverse events during rehabilitation training, and these are summarized in Table 2.

**Table 2:** Exclusion criteria for the cardiac rehabilitation program

∙ Cognitive impairment or mental disorder identified by MMSE score < 24	
∙ Unstable angina or onset of myocardial infarction within < 2 weeks	
∙ Uncontrolled serious arrhythmia	
∙ Uncontrolled hypertension (resting systolic blood pressure > 160 mmHg or resting diastolic blood pressure > 100 mmHg)	
∙ Cardiac function class IV	
∙ Coronary heart disease accompanied by acute complications (coronary artery dissection, ventricular aneurysm, large area of myocardial infarction associated with shock, acute vascular occlusion including stent thrombosis) and high risk of cardiovascular events posed by rehabilitation exercise	
∙ Active pericarditis or myocarditis, serious infection, chronic obstructive pulmonary disease, moderate to serious aortic stenosis, resting heart rate after drug control > 100 times/min	
∙ New deep vein thrombosis, thrombophlebitis, aortic dissection or aneurysm in other parts of the body, lower limb occlusive atherosclerosis	
∙ Inability to tolerate exercise due to fracture, arthritis or muscle pain	
∙ Abnormal electrolytes such as potassium, sodium or calcium	
∙ Uncontrolled hyperthyroidism or hypothyroidism at the time of recruitment	
∙ COPD with signs of infection such as fever, sore throat, coughing, etc.	

*COPD* chronic obstructive pulmonary disease, *MMSE* mini-mental state examination

### Randomization

**Fig. 1:**
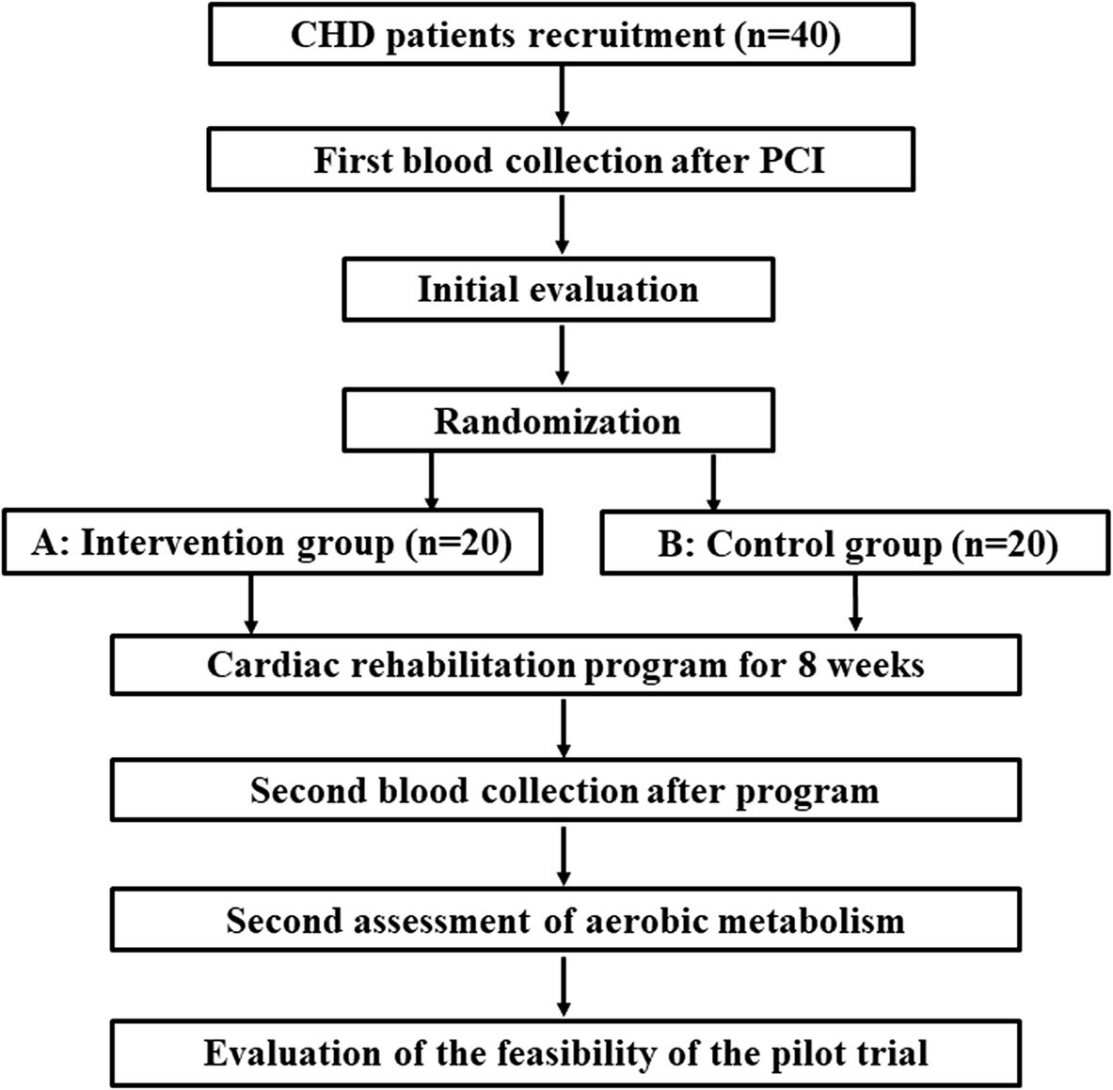
Study flow chart. Eligible participants with coronary heart disease (CHD) after percutaneous coronary intervention (PCI) will be randomly allocated to the intervention or control group. The exercise intervention is an outpatient clinical rehabilitation program with targeted intensity, which includes a 30-min session three times a week for 8 weeks. Participants in the control group will be prescribed the same number of exercise sessions without targeted intensity. Cardiopulmonary exercise testing will be performed before and after the 8-week cardiac rehabilitation program to assess changes in cardiopulmonary function

Participants will be randomly allocated (1:1 ratio) to either the intervention or control group once screening assessments are completed and eligibility for the study is confirmed (Fig. 1). A research coordinator will generate the random allocation sequence, enroll participants, and assign participants to interventions. A stratified randomization scheme based on gender will be used. Microsoft Excel Formula Rand will be used to allocate patients to the intervention or control group. A random number between 0 and 1 will be generated for each patient; if the last digit is an even number the patient will be assigned to group A (intervention) and if the last digit is an odd number the patient will be assigned to group B (control) (Table 3). Forty participants will be allocated randomly to group A (intervention exercise group with targeted intensity, *n* = 20) or B (control group with leisure exercise, *n* = 20).

**Table 3:** A stratified randomization scheme based on gender

Male			Female		
Serial number	Random number	Group	Serial number	Random number	Group
1	0.699718	A	1	0.299478	A
2	0.474505	B	2	0.228082	A
3	0.278238	A	3	0.905825	B
4	0.063581	B	4	0.288985	B
5	0.543526	A	5	0.204267	B
6	0.645542	A	6	0.004536	A
7	0.606382	A	7	0.876704	A
8	0.358782	A	8	0.765598	A
9	0.913533	B	9	0.989875	B
10	0.394207	B	10	0.735247	B
11	0.101518	A	11	0.968638	A
12	0.830019	B	12	0.975038	A
13	0.454501	B	13	0.355564	A
14	0.941391	B	14	0.730377	B
15	0.748873	B	15	0.087323	B
16	0.736089	B	16	0.318215	B
17	0.026114	A	17	0.794593	B
18	0.294124	A	18	0.322396	A
19	0.801002	A	19	0.180908	A
20	0.448855	B	20	0.552161	B

Microsoft Excel Rand is performed to generate random numbers between 0 and 1, last digit even number for group A (intervention) and odd for group B (control). Patients are then allocated to intervention and control groups chronologically

### Sample size determination

Given the pilot nature of this trial, we will focus on the feasibility of the study and ensure that the exercise intervention runs smoothly, we will not calculate a sample size to determine the power in this study [20]. However, it is recommended that a minimum of 30 participants is required to achieve sufficient precision to enable sample size calculation for subsequent studies [21]. Therefore, we plan to recruit 40 participants, which will allow the completion of data collection from 30 subjects, with a 75% participant retention rate.

### Study procedures

#### Telephone follow up before exercise intervention

Eligible patients who are willing to participate in our CR program will be contacted by a telephone call 1–2 weeks after PCI. Patients in a stable condition will be recommended to start outpatient rehabilitation in our clinic as soon as possible. A “stable condition” is determined by the following: the participant self-reports satisfaction over the phone and denies recent chest pain or any other disorders that need additional medications; the physician confirms that the patient’s vital signs are stable and within normal limits at the outpatient clinic, for example, resting heart rate < 100/min, resting systolic blood pressure < 140 mmHg and diastolic blood pressure <90 mmHg. If an individual is not considered stable, there is an additional waiting period of 2 weeks. That person will be checked again 2 weeks later to determine if he/she is sufficiently stable to participate in the CR program.

#### Exercise prescription

All participants will undergo cardiopulmonary exercise testing (CPET) to obtain initial parameters of anaerobic threshold (AnT), which is defined as the highest sustained intensity of exercise for which measurement of oxygen uptake by means of CPET can account for the entire energy requirement, on the first day of the CR program. The targeted intensity is determined based on the heart rate recorded 1 min before AnT during CPET to provide an individualized CR training prescription for each participant in the intervention group. Thus, exercise intensity will not be affected by medications because the intensity is determined on an individual basis. Participants in the control group will undergo the same number of exercise sessions without targeted intensity. The CR program also includes CHD management strategies such as concomitant therapy, balanced diet nutrition, psychosocial consultation, and smoking cessation. There are no differences in disease management between the two groups.

#### Exercise intervention

The exercise intervention is an outpatient clinical rehabilitation program delivered one-on-one to individuals in a 30-min session and three times a week for 8 weeks in the hospital outpatient clinic. The 30-min CR procedure includes 5 min of warm-up exercise, 20 min of cycle ergometer (Ergoline GmbH, Germany) exercise with targeted intensity, and 5 min of cool-down exercise. The targeted intensity is set as the heart rate 1 min before AnT based on CPET. We expect that exercise parameters will improve gradually as individuals reach their targeted intensity more frequently with time over the 8 weeks. Participants are closely monitored using a heart rate monitoring device connected to the cycle ergometer and will be guided by the CR team, which consists of cardiologists, rehabilitation physicians, physiotherapists, nurses, and clinical research coordinators. The research coordinator will record the attendance of each participant and will ask the participant to come on the weekend to compensate for any missed sessions. In addition, the CR protocol can be extended to accommodate an intervening illness. Nevertheless, the participant is allowed to quit the program at any time if he/she wants to do so. Participants will be followed closely to ensure adherence. All CR providers are trained to follow the same protocol to ensure intervention fidelity. Participants in the control arm will receive telephone calls once a week to monitor their physical conditions and will be encouraged to take a walk at least 30 min at a time and three times a week during the 8-week study at home but not at the program center. The information for the control group will be recorded using activity tracking apps such as activity tracker bands, watches and smartphones so that the authors will know how active the control-arm participants are. The second CPET will be conducted after the 8-week CR program. Graduates will also be advised to take concomitant medications, continue engaging in appropriate exercise, eat a balanced diet, and quit smoking.

#### Telephone follow up after 3 months

Participants in both groups will receive follow-up calls 3 months after the end of the program to provide information on their most updated health condition such as smoking status, cardiac risk control medication (aspirin, statins, beta-blockers, etc.), diet, psychological status, and physical activity. At the end of the CR session, patients in both the intervention and control groups will be prescribed a home exercise program using community-based rehabilitation facilities and will be encouraged to live a more active lifestyle.

### Data collection

**Fig. 2:**
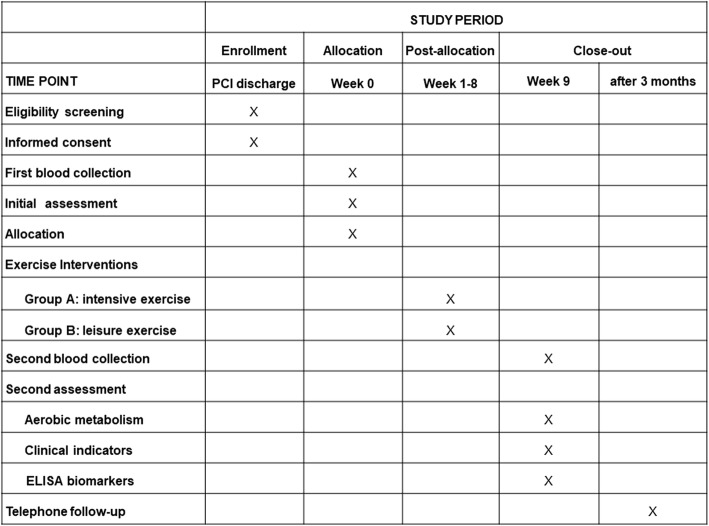
Standard Protocol Items: Recommendations for Interventional Trials (SPIRIT) timeline of measurements. Recommended content for the schedule of enrollment, intervention, and assessments. ELISA, enzyme linked immunosorbent assay; PCI, percutaneous coronary intervention

Assessors are trained to collect trial data from the outpatient clinic and laboratory. The following data will be collected from all participants before and after the rehabilitation training as shown in the SPIRIT diagram (Fig. 2): the aerobic metabolism parameter (AnT), clinical indicators (blood pressure, heart rate, body mass index, smoking status, and comorbidities), laboratory indicators (total cholesterol, high-density lipids (HDL)-cholesterol, low-density lipids (LDL)-cholesterol, triacylglycerides, apolipoproteins, homocysteine, glucose, etc.), and blood samples for additional biomarkers (C-reactive protein, macrophage inflammatory protein-1α, pentraxin-3, monocyte chemotactic protein-1, interleukins, transforming growth factor-β, interferon-γ, brain natriuretic peptide, growth differentiation factor-15, vascular endothelial growth factor-B, myeloperoxidase, adiponectin, matrix metalloproteinase-1, 2, or 9, matrix metalloproteinases tissue inhibitor-1, phosphodiesterase-9A). Data will be collected at 3 months to assess the short-term prognosis and risk factors for cardiac adverse events after the CR program.

### Outcome measurements

The primary outcome is the feasibility of the trial. Recruitment rate and retention rate as feasibility parameters will be analyzed. The secondary outcomes are aerobic metabolism parameters and clinical and laboratory indicators that will be used to identify cardiac risk factors such as hypertension, dyslipidemia, diabetes mellitus, obesity, and smoking status. The exploratory outcomes are additional biomarkers such as inflammatory markers (C-reactive protein, macrophage inflammatory protein-1α, pentraxin-3, monocyte chemotactic protein-1, interleukins, transforming growth factor β), an immune-modulatory marker (interferon-γ), myocyte stress markers (brain natriuretic peptide and growth differentiation factor-15), a left ventricular dysfunction marker (vascular endothelial growth factor-B), an oxidative stress marker (myeloperoxidase), a metabolic hormone (adiponectin), extracellular-matrix remodeling markers (matrix metalloproteinase-1, 2, 9, and matrix metalloproteinases tissue inhibitor-1), and a signal transduction regulator (phosphodiesterase-9A) to further evaluate a patient’s underlying cardiovascular function at the molecular level.

### Data assessment

The objective of this pilot study is to test the feasibility of recruitment, retention, intervention, and collection of data on aerobic metabolism parameters and clinical and laboratory measurements at the same time. Mean changes in outcomes over time will be summarized graphically and descriptively within each group. These changes will be compared between groups and will be described with associated 95% confidence intervals to explore possible effects of the intervention, recognizing that this pilot trial is not powered to detect clinically meaningful effects. Feasibility parameters, such as recruitment rate and retention rate, will be analyzed to determine whether it is worth continuing to conduct a large-scale trial that builds from the current pilot trial. The key feasibility criteria include recruitment of 40 participants, attainment of a 75% participant retention rate, and measurement of all outcomes in 90% of all participants. These parameters will be assessed at the end of the pilot trial by a statistician who is not involved in the study.

## Discussion

The benefits of CR are well-recognized in America and Europe and are widely recommended by the American Association of Cardiovascular and Pulmonary Rehabilitation and European Society of Cardiology as secondary prevention in patients with coronary disease [7, 22, 23]. However, the CR program is far underdeveloped and in China has thereby resulted in a high rate of recurrence of heart attacks and adverse cardiovascular events [24]. China faces an aging population with an increasing lifespan, which will result in a large proportion of people suffering from cardiovascular events in the near future. Therefore, increasing awareness of the benefits of CR, improving CR availability throughout China, and providing safe and effective CR programs for Chinese patients with CHD are extremely important.

To our knowledge, the present pilot trial is the first clinical study that comprehensively screens multiple blood biomarkers to explore the potential underlying molecular mechanisms and changes in cardiovascular function after aerobic exercise-based CR training. Results of this pilot study can be used to predict the feasibility and operational acceptability of a future large-scale pivotal trial that promotes the generalization of the CR program in China.

However, this pilot trial has several limitations. Given the large age range of subjects from 45 to 80 years and the relatively small sample size after allocation to subgroups (male and female), physiology and biomarker profiles can vary greatly. The second limitation is the disparate modes between the intervention (cycle ergometer) and control (walk) groups. Specifically, the intervention group receives cycle ergometer training for exercise safety and to prevent patients from falling during treadmill training whereas the control arm does not receive this training. Moreover, whereas all of the intervention participants will be monitored with a standardized heart rate monitor during each intervention session, activity in the control arm will not be monitored using the same tool and will instead be monitored by whatever tracking app the participant has available. As a consequence, the data quality may be of very low quality or missing in the control arm. The third limitation is that outcome assessment of metabolism and clinical indicators (blood pressure, heart rate etc.) is not blinded. As a consequence, the quantitative results of the trial may not be completely reliable as there could be different quality of assessments in the two groups of the trial. However, a statistician who is not involved in the study will be invited to assess the data according to a pre-specified analysis plan, and the groups will be labeled A and B, to ensure that the data are analyzed blind. In a future larger trial, procedures will be put in place to ensure blinding of outcome assessors to avoid bias. The feasibility measures are the focus of the current pilot study and these will not be subject to potential observer bias.

### Trial status

This is an ongoing trial. We have not completed patient recruitment at the time of submission. So far, 13 patients have been enrolled, with 6 in the intervention group and 7 in the control group.

## Additional files

Additional file 1: SPIRIT checklist. (DOCX 38 kb)

Additional file 2: CONSORT checklist. (DOC 226 kb)

Additional file 3: Informed consent. (DOCX 26 kb)

## Abbreviations

AnT: Anaerobic threshold
CHD: Coronary heart disease
CONSORT: Consolidated Standards of Reporting Trials
CPET: Cardiopulmonary exercise testing
CR: Cardiac rehabilitation
CVD: Cardiovascular disease
PCI: Percutaneous coronary intervention
SPIRIT: Standard Protocol Items: Recommendations for Interventional Trials
