# Supplementary material for: Aerobic exercise-based cardiac rehabilitation in Chinese patients with coronary heart disease: study protocol for a pilot randomized controlled trial
Source: Trials. 2018 Jul 9;19:363. doi: 10.1186/s13063-018-2771-8 (PMC6038344; doi:10.1186/s13063-018-2771-8)
Supplement: Supplementary file 1 — SPIRIT checklist. (DOCX 38 kb) [file 13063_2018_2771_MOESM1_ESM.docx]

**Additional file 1: SPIRIT Checklist: Recommended Items to Address in a Clinical Trial Protocol and Related Documents**

| **Section/Item**  **Administrative information** | **Item Number** | **Description** |
| --- | --- | --- |
| Title | 1 | Descriptive title identifying the study design, population, interventions, and, if applicable, trial acronym  Aerobic exercise-based cardiac rehabilitation in Chinese patients with coronary heart disease: study protocol for a pilot randomized controlled trial |
| Trial registration | 2a | Trial identifier and registry name. If not yet registered, name of intended registry.  Trial identifier：Chinese Clinical Trial Registry (ChiCTR-IPR-17010556)  Registry name: A randomized controlled trial to assess feasibility of aerobic exercise-based cardiac rehabilitation on patients with coronary heart disease |
|  | 2b | All items from the World Health Organization Trial Registration Data Set (Appendix Table, available at [www.annals.org](http://www.annals.org))  Not applicable for this pilot trial. |
| Protocol version | 3 | Date and version identifier  Date Oct 22, 2017 and version 2.0 |
| Funding | 4 | Sources and types of financial, material, and other support  This work was supported by Shanghai Municipal Commission of Health and Family Planning (grants ZK2015A29 and ZK2015A30) and National Natural Science Foundation of China (grant number 81672260). |
| Roles and responsibilities | 5a | Names, affiliations, and roles of protocol contributors  Richard Y. Cao, Qiongyao Mi, Qing Li, Wenchao Yuan, Yueyou Ding, Hongchao Zheng, Jian Yang: The Joint Laboratory of Cardiac Rehabilitation, Shanghai University & Shanghai Xuhui Central Hospita;  Authors' roles: RYC, JY and HZ conceived this study and led its design and coordination. QM and RC drafted the manuscript and developed the randomization scheme. QL participated in study design and coordinated the study. WY and YD assisted in coordinating the study. RYC and JY help to edit the manuscript. All authors have read and approved the final manuscript. |
|  | 5b | Name and contact information for the trial sponsor  Shanghai Municipal Commission of Health and Family Planning, 193 Hankou Road, Huangpu District, Shanghai 200002, China  National Natural Science Foundation of China, 83 Shuangqing Road, Haidian District, Beijing 100085, China |
|  | 5c | Role of study sponsor and funders, if any, in study design; collection, management, analysis, and interpretation of data; writing of the report; and the decision to submit the report for publication, including whether they will have ultimate authority over any of these activities  Not applicable for this pilot trial. |
|  | 5d | Composition, roles, and responsibilities of the coordinating center, steering committee, end point adjudication committee, data management team, and other individuals or groups overseeing the trial, if applicable (see item 21a for DMC)  Not applicable for this pilot trial. |
| **Introduction** | | |
| Background and rationale | 6a | Description of research question and justification for undertaking the trial, including summary of relevant studies (published and unpublished) examining benefits and harms for each intervention  Cardiovascular disease is the leading cause of morbidity and mortality in the world and in China. Cardiac rehabilitation (CR) has been demonstrated beneficial to reduce cardiovascular mortality, myocardial infarction, and cerebrovascular events. This pilot study seeks to address aspects of feasibility and key issues of uncertainty of aerobic exercise-based CR program on patients with coronary heart disease (CHD) in China. |
|  | 6b | Explanation for choice of comparators  This is the first prospective randomized controlled study in China to assess parameters of aerobic metabolism and cardiac risk factors, as well as to screen substantial blood biomarkers comprehensively to reveal underlying molecular mechanisms of cardiovascular function changes after aerobic exercise with targeted intensity in CHD participants. We will compare parameters between patients in intensive exercise and leisure exercise groups. |
| Objectives | 7 | Specific objectives or hypotheses  The purpose of this pilot study is to test the feasibility of aerobic exercise-based CR on parameters of aerobic metabolism capacity, cardiac risk factors, and underlying cardiovascular function expressed by molecular biomarkers in patients with CHD after PCI. |
| Trial design | 8 | Description of trial design, including type of trial (e.g., parallel group, crossover, factorial, single group), allocation ratio, and framework (e.g., superiority, equivalence, noninferiority, exploratory)  This is a single center prospective parallel randomized controlled study carried out at a regional hospital in Shanghai. Forty CHD patients after percutaneous coronary intervention will be randomly divided 1:1 into the intervention or control group. |
| **Methods** | | |
| Participants, interventions, and outcomes | | |
| Study setting | 9 | Description of study settings (e.g., community clinic, academic hospital) and list of countries where data will be collected. Reference to where list of study sites can be obtained  This single center clinical study will be carried out in Shanghai Xuhui Central Hospital located at 966 Middle Huaihai Road, Shanghai 200031. |
| Eligibility criteria | 10 | Inclusion and exclusion criteria for participants. If applicable, eligibility criteria for study centers and individuals who will perform the interventions (e.g., surgeons, psychotherapists)  Inclusion criteria are (1) diagnosed to be CHD by a cardiologist based on combined evidences of clinical symptoms and tests of electrocardiogram and/or echocardiogram and/or coronary angiogram; (2) aged between 45-80 years old; (3) classified as low to moderate cardiac risk for CR based on Chinese experts’ cardiac risk stratification; (4) signed consent form.  Exclusion criteria are (1) cognitive impairment or mental disorder assessed by MMSE < 24; (2) unstable angina or myocardial infarction onset < 2 weeks; (3) uncontrolled serious arrhythmia; (4) uncontrolled hypertension (resting systolic blood pressure > 160 mmHg or resting diastolic blood pressure > 100 mmHg); (5) cardiac function class IV; (6) coronary heart disease accompanied by acute complications (coronary artery dissection, ventricular aneurysm, large area of myocardial infarction associated with shock, acute vascular occlusion including stent thrombosis) and high cardiac risk for rehabilitation; (7) active pericarditis or myocarditis, serious infection, chronic obstructive pulmonary disease, moderate to serious aortic stenosis, resting heart rate after drug control > 100 times/min (8)new deep vein thrombosis, thrombophlebitis, aortic dissection or aneurysm in other parts of the body, lower limb occlusive atherosclerosis; (9) cannot tolerate exercise with fracture, arthritis or muscle pain; (10) abnormal electrolyte such as potassium, sodium or calcium; (11) uncontrolled hyperthyroidism or hypothyroidism at the time of recruitment; (12) **COPD** with signs of infection such as fever, sore throat, coughing. |
| Interventions | 11a | Interventions for each group with sufficient detail to allow replication, including how and when they will be administered  Eligible patients who are willing to participate in our CR program will be followed up by a phone call 1-2 weeks after PCI. Patients in stable condition will be recommended to start outpatient rehabilitation in our clinic as soon as possible. The exercise intervention is an outpatient clinical rehabilitation program delivered one-on-one to individuals with 30 minutes each time, 3 times a week for 8-week duration in the hospital. The 30 minutes CR procedure includes 5 minutes warming-up exercise, 20 minutes ergometer cycle exercise with targeted intensity, and 5 minutes cooling-down exercise. The targeted intensity is set as the heart rate 1 min before anaerobic threshold based on cardiopulmonary exercise testing (CPET). Participants in control arm will receive telephone contact to monitor their physical condition and be encouraged to take a walk at least 30 minutes each time, 3 times a week during the 8-week study at home. |
|  | 11b | Criteria for discontinuing or modifying allocated interventions for a given trial participant (e.g., drug dose change in response to harms, participant request, or improving/worsening disease)  If a participant encounters abnormal vital signs, he/she should stop CR to see a doctor. |
|  | 11c | Strategies to improve adherence to intervention protocols, and any procedures for monitoring adherence (e.g., drug tablet return, laboratory tests)  Frequent follow-up phone call is an important aspect for monitoring adherence. |
|  | 11d | Relevant concomitant care and interventions that are permitted or prohibited during the trial  Concomitant medications such as aspirin, angiotensin converting enzyme inhibitors, statins are permitted. |
| Outcomes | 12 | Primary, secondary, and other outcomes, including the specific measurement variable (e.g., systolic blood pressure), analysis metric (e.g., change from baseline, final value, time to event), method of aggregation (e.g., median, proportion), and time point for each outcome. Explanation of the clinical relevance of chosen efficacy and harm outcomes is strongly recommended  The primary outcome is the feasibility of the trial. Recruitment rate and retention rate as feasibility parameters will be analyzed. The secondary outcomes are aerobic metabolism parameters as well as clinical and laboratory indicators that will be used to identify cardiac risk factors such as hypertension, dyslipidemia, diabetes, obesity, and smoking status. The exploratory outcomes are additional biomarkers such as inflammatory markers (C-reactive protein, macrophage inflammatory protein-1α, Pentraxin-3, monocyte chemotactic protein-1, interleukins, transforming growth factor-β), an immune-modulatory marker (interferon-γ), myocyte stress markers (brain natriuretic peptide and growth differentiation factor-15), a left ventricular dysfunction marker (vascular endothelial growth factor B), an oxidative stress marker (myeloperoxidase), a metabolic hormone (adiponectin), extracellular-matrix remodeling markers (matrix metalloproteinase-1, 2, 9, and matrix metalloproteinases tissue inhibitor-1), and a signal transduction regulator (phosphodiesterase-9A) to further evaluate a patient’s underlying cardiovascular function at the molecular level. |
| Participant timeline | 13 | Time schedule of enrollment, interventions (including any runins and washouts), assessments, and visits for participants. A schematic diagram is highly recommended (Figure).  SPIRIT timeline of measurements is shown in Figure 2. |
| Sample size | 14 | Estimated number of participants needed to achieve study objectives and how it was determined, including clinical and statistical assumptions supporting any sample size calculations  Not applicable for this pilot trial. |
| Recruitment | 15 | Strategies for achieving adequate participant enrollment to reach target sample size  Close collaboration between physicians in Cardiology and Rehabilitation Departments. |
| Assignment of interventions (for controlled trials) | | |
| Allocation Sequence generation | 16a | Method of generating the allocation sequence (e.g., computer-generated random numbers), and list of any factors for stratification. To reduce predictability of a random sequence, details of any planned restriction (e.g., blocking) should be provided in a separate document that is unavailable to those who enroll participants or assign interventions.  A stratified randomization scheme based on gender (M=male and F=female) is used. Microsoft Excel formula Rand will be used to generate a random number even for Group A (intervention) and odd for Group B (control) to allocate patients to intervention or control group. Each group will have 20 participants. |
| Allocation concealment mechanism | 16b | Mechanism of implementing the allocation sequence (e.g., central telephone; sequentially numbered, opaque, sealed envelopes), describing any steps to conceal the sequence until interventions are assigned  Allocation will be sequentially numbered. |
| Implementation | 16c | Who will generate the allocation sequence, who will enroll participants, and who will assign participants to interventions  Qiongyao Mi and Richard Y. Cao |
| Blinding (masking) | 17a | Who will be blinded after assignment to interventions (e.g., trial participants, care providers, outcome assessors, data analysts), and how  This pilot trial is not blinded. |
|  | 17b | If blinded, circumstances under which unblinding is permissible, and procedure for revealing a participant’s allocated intervention during the trial  Not applicable for this pilot trial. |
| Data collection, management, and analysis | | |
| Data collection methods | 18a | Plans for assessment and collection of outcome, baseline, and other trial data, including any related processes to promote data quality (e.g., duplicate measurements, training of assessors) and a description of study instruments (e.g., questionnaires, laboratory tests) along with their reliability and validity, if known. Reference to where data collection forms can be found, if not in the protocol.  Assessors are trained to collect trial data from the outpatient clinic and laboratory before and after the rehabilitation program. Molecular biomarkers will be measured triplicated to promote quality.  Data collection forms will be kept in the hospital research office. |
|  | 18b | Plans to promote participant retention and complete follow-up, including list of any outcome data to be collected for participants who discontinue or deviate from intervention protocols  Frequent follow-up phone call will be conducted to promote participant retention. |
| Data management | 19 | Plans for data entry, coding, security, and storage, including any related processes to promote data quality (e.g., double data entry; range checks for data values). Reference to where details of data management procedures can be found, if not in the protocol.  Data entry will be conducted by Qiongyao Mi and monitored by Qing Li to promote data quality. |
| Statistical methods | 20a | Statistical methods for analyzing primary and secondary outcomes. Reference to where other details of the statistical analysis plan can be found, if not in the protocol.  Mean changes in outcomes over time will be summarized graphically and descriptively within each group. These changes will be compared between groups and will be presented with associated 95% confidence intervals to explore possible effects of the intervention, whilst recognizing that this pilot trial study is not powered to detect clinically meaningful effects. |
|  | 20b | Methods for any additional analyses (e.g., subgroup and adjusted analyses)  Not applicable for this pilot trial. |
|  | 20c | Definition of analysis population relating to protocol nonadherence (e.g., as-randomized analysis), and any statistical methods to handle missing data (e.g., multiple imputation)  Not applicable for this pilot trial. |
| Monitoring | | |
| Data monitoring | 21a | Composition of DMC; summary of its role and reporting structure; statement of whether it is independent from the sponsor and competing interests; and reference to where further details about its charter can be found, if not in the protocol. Alternatively, an explanation of why a DMC is not needed.  Not applicable for this pilot trial. |
|  | 21b | Description of any interim analyses and stopping guidelines, including who will have access to these interim results and make the final decision to terminate the trial  Not applicable for this pilot trial. |
| Harms | 22 | Plans for collecting, assessing, reporting, and managing solicited and spontaneously reported adverse events and other unintended effects of trial interventions or trial conduct  Not applicable for this pilot trial. |
| Auditing | 23 | Frequency and procedures for auditing trial conduct, if any, and whether the process will be independent from investigators and the sponsor  Not applicable for this pilot trial. |
| **Ethics and dissemination** | | |
| Research ethics approval | 24 | Plans for seeking REC/IRB approval  Ethics Committee of Shanghai Xuhui Central Hospital (Approval No. 2016-10) |
| Protocol amendments | 25 | Plans for communicating important protocol modifications (e.g., changes to eligibility criteria, outcomes, analyses) to relevant parties (e.g., investigators, RECs/IRBs, trial participants, trial registries, journals, regulators)  If there are changes to eligibility criteria, outcomes, analyses, a new version of protocol will be submitted to Ethics Committee of Shanghai Xuhui Central Hospital for approval. |
| Consent or assent | 26a | Who will obtain informed consent or assent from potential trial participants or authorized surrogates, and how (see item 32)  Qiongyao Mi will obtain informed consent from potential trial participants. |
|  | 26b | Additional consent provisions for collection and use of participant data and biological specimens in ancillary studies, if applicable  Not applicable for this pilot trial. |
| Confidentiality | 27 | How personal information about potential and enrolled participants will be collected, shared, and maintained in order to protect confidentiality before, during, and after the trial  Participants’ personal information will be kept confidentiality the same way as their medical histories in the hospital before, during, and after the trial. |
| Declaration of interests | 28 | Financial and other competing interests for principal investigators for the overall trial and each study site  No |
| Access to data | 29 | Statement of who will have access to the final trial data set, and disclosure of contractual agreements that limit such access for investigators  Not applicable |
| Ancillary and post-trial care | 30 | Provisions, if any, for ancillary and post-trial care, and for compensation to those who suffer harm from trial participation  Not applicable |
| Dissemination policy | 31a | Plans for investigators and sponsor to communicate trial results to participants, health care professionals, the public, and other relevant groups (e.g., via publication, reporting in results databases, or other data-sharing arrangements), including any publication restrictions  Not applicable |
|  | 31b | Authorship eligibility guidelines and any intended use of professional writers  Not applicable |
|  | 31c | Plans, if any, for granting public access to the full protocol, participant-level data set, and statistical code  Not applicable |
| **Appendices** | | |
| Informed consent materials | 32 | Model consent form and other related documentation given to participants and authorized surrogates  Attached in Appendix3. |
| Biological specimens | 33 | Plans for collection, laboratory evaluation, and storage of biological specimens for genetic or molecular analysis in the current trial and for future use in ancillary studies, if applicable  Blood samples from participants will be collected and stored at -80^0^C until plasma samples were analyzed by the end of the study. |
