# Supplementary material for: Aerobic exercise-based cardiac rehabilitation in Chinese patients with coronary heart disease: study protocol for a pilot randomized controlled trial
Source: Trials. 2018 Jul 9;19:363. doi: 10.1186/s13063-018-2771-8 (PMC6038344; doi:10.1186/s13063-018-2771-8)
Supplement: Supplementary file 3 — Informed consent. (DOCX 26 kb) [file 13063_2018_2771_MOESM3_ESM.docx]

**Informed Consent**

Dear Sir/Madam:

Welcome to the Department of Rehabilitation. You are diagnosed as coronary heart disease by your physician based on combined evidences of clinical symptoms and tests of electrocardiogram and coronary angiogram. We would like to invite you to attend a aerobic exercise-based cardiac rehabilitation, which has been demonstrated beneficial for coronary patients to reduce cardiovascular mortality, myocardial infarction, and cerebrovascular events. This trial is supported by Shanghai Municipal Commission of Health and Family Planning (grant number ZK2015A40), National Natural Science Foundation of China (grant number 81672260) and has been approved by Shanghai Xuhui Central Hospital Ethics Committee.

In order to have a better understanding of the study, please read the following items carefully. You may also consult your doctor or communicate with your relatives.

**1. Introduction of the study**

**1.1 Background**

Cardiac rehabilitation, a multifaceted intervention program, has been demonstrated to show great benefits to reduce cardiovascular mortality, myocardial infarction, and cerebrovascular events. It is designed to assist cardiac patients to achieve optimal physical, psychological and social status, in order to stabilize, slow, or even reverse the progression of the underlying atherosclerotic processes, thereby reducing morbidity and mortality.

**1.2 Aim**

This research is to explore the changes in clinical and laboratory indicators before and after the aerobic exercise rehabilitation, and to assess the feasibility of aerobic exercise rehabilitation.

**1.3 Processes**

This study will be carried out in Shanghai Xuhui Central Hospital. We are going to recruit 60 patients. Inclusion criteria are (1) diagnosed to be CHD by a cardiologist based on combined evidences of clinical symptoms and tests of electrocardiogram and/or echocardiogram and/or coronary angiogram; (2) aged between 45-80 years old; classified as low to moderate cardiac risk for CR based on Chinese experts’ cardiac risk stratification; (4) signed consent form. All participants will take the first cardiopulmonary exercise testing to obtain initial parameters of anaerobic threshold. Take the heart rate of 1 minute before anaerobic threshold as targeted intensity of individualized training for participants in group A. Participants in group B will be prescribed with leisure exercise without targeted intensity. The exercise intervention is an outpatient clinical rehabilitation program with 30 minutes each time, 3 times a week for 8-week duration. The 30 minutes CR procedure includes 5 minutes warming-up exercise, 20 minutes ergometer cycle exercise with targeted intensity, and 5 minutes cooling-down exercise. Participants are closely watched via heart rate monitoring device and guided by the CR team consisting of cardiologists, rehabilitation physician, physiotherapists, nurses and clinical research coordinators. Participants in control group will be encourage to take a walk at least 30 minutes each time, 3 times a week during the 8-week study. The second cardiopulmonary exercise testing will be conducted after the 8-week CR program. All entrants will be advised to take concomitant medications, quit smoking, accept nutritional guidance and psychological consultation.

**2. What will happen after enrolment?**

(1) The doctor will check your medical history to collect the clinical data such as your age, heart rate, blood pressure, BMI as well as cardiopulmonary exercise testing in initial and final evaluation.

(2) You will be randomly divided into target intensity aerobic exercise group (group A) and leisure exercise (group B). The exercise intervention duration is 8 weeks. Five ml blood will be collected before and after the intervention.

**3. What will you be beneficial from this study?**

This study will enable you to enjoy a set of rehabilitation training (including free psychological counselling, nutritional guidance and so on). After the study you will get 300 yuan as outpatient follow-up fee.

**4. Related fees**

You don’t need to pay any fees during this study.

**5. Possible risks and inconvenience**

**5.1** Risks: Possible risks of rehabilitation training are (1) As you are randomly allocated into different groups, the outcome may differ; (2) On the basis of the original coronary artery disease, the patient situation might be worsen in some extreme cases; (3) Accidental falls; (4) Other unforeseen factors. Therefore, if you feel uncomfortable please let us know as soon as possible. We will give you proper medical care.

**5.2** Inconvenience: This study will occupy your time or influence your daily life during the 8-week period.

**6. Your rights**

(1) Confidential personal information. Your records will be placed in the hospital confidentially. Any public report related to the study results will not disclose your personal identity.

(2) You can ask any questions about this study at any time. If there is any changes in the research process which may affect your willingness to continue participating in the study, the doctor will inform you immediately.

(3) Whether or not to continue the program is totally up to you.

**7. Now what to do?**

(1) Ask us questions about the study.

(2) If there are no more questions, tell us your decision.

Thanks for reading the above information. If you would like to join in this study, please sign the Informed consent.

**Signature page**

**Study title**：A randomized controlled pilot trial to assess feasibility of aerobic

exercise-based cardiac rehabilitation on patients with coronary heart disease

**Implementation unit:** Shanghai Xuhui Central Hospital

**Declaration of Consent**

I have read the above information. I have had the opportunity to ask questions about it and all questions have been answered to my satisfaction. I consent voluntarily to participate in this research.

**Signature of Participant** ___________________

**Date:** ___________________

**Telephone**：___________________

I confirm that I have explained the details of this test to the patient, including his/her benefits and possible risks，and give him/her a copy of informed consent.

**Name of researcher**：___________________

**Date**: ___________________

**Telephone**：___________________

The contact person at Shanghai Xuhui Central Hospital Ethics Committee：Yanyan Wang

Tel：021-54043676
